# Neuropharmacological Aspects of Cognitive Neurorehabilitation in Epilepsy

**DOI:** 10.1155/2006/893053

**Published:** 2006-05-11

**Authors:** Marco Mula, Michael R. Trimble

**Affiliations:** ^1^Department of NeurologyAmedeo Avogadro UniversityNovaraItaly; ^2^Institute of NeurologyUniversity College LondonQueen SquareLondonUK

## Abstract

The role of CNS neuromodulators in cognitive neurorehabilitation can be related to two main issues: (1) the negative impact on cognition of drug categories prescribed for different neurologic symptoms, such as spasticity, extrapyramidal symptoms, or epileptic seizures; (2) their possible role in neuroprotection and amelioration of the cognitive status of the patient, especially attention and memory. This paper reviews different pharmacological aspects of cognitive neurorehabilitation in epilepsy.

When approaching the role of central nervous system (CNS) drugs in cognitive neurorehabilitation, one must consider two main aspects: 1) the negative impact on cognition of different drug categories; 2) the pharmacological instruments available for amelioration of cognitive deficits. In this paper, as we consider epilepsy and its management, we first make some relevant comments on neurotransmitter relationships to cognition.

## 1. Neurotransmitters and cognition

From a psychopharmacological point of view, two cognitive domains seem of most importance, namely attention and memory. Current evidence suggests that the former is interlinked with the dopamine/norepinephrine systems, while the latter is dependent on the cholinergic system [41]. In clinical practice, the most commonly used agents to enhance attention are methylphenidate and d-amphetamine. Pemoline and metamphetamine are also available in some countries, but are not as widely used, because of their liver toxicity and abuse potential respectively. Both methylphenidate and d-amphetamine mainly determine release of dopamine from presynaptic terminals, but they also inhibit the dopamine transporter, and in some models they actually reverse its direction, leading to further dopamine release from the synapse. Methylphenidate is less potent as a dopamine releaser, but is longer acting than d-amphetamine. Another form of amphetamine, l-amphetamine, releases norepinephrine as well as dopamine through a similar mechanism and, in clinical practice, a mixture of d,l-amphetamine is usually prescribed.

Memory is one of the most complex functions of the human brain and involves many neurotransmitters and cortical-subcortical neuronal networks. However, we know that specific disorders of the cholinergic system are characterized by memory disturbances and the therapeutic agents currently available, that can improve memory function, usually act on this system in some way or other. The major cholinergic strategies for enhancing memory are cholinesterase inhibition by a number of agents that are clinically available, including donepezil, tacrine, rivastigmine, metrifonate, physostigmine and galantamine. They represent an important class of drugs now used widely in patients with Alzheimer disease and other dementias, but they may also find use in other neurological settings.

We now look at investigations in patients with epilepsy based upon these theoretical foundations.

ISSN 0953-4180/06/$17.00 © 2006 – IOS Press and the authors. All rights reserved

## 2. Epilepsy, cognition and antiepileptic drugs

There is a considerable literature which suggests that certain antiepileptic drugs and in particular polytherapy markedly affect cognitive function in epilepsy. This conclusion emerged from earlier studies which revealed that many patients with epilepsy were treated with two or more, often sedative, drugs and that tailoring therapy from polytherapy to monotherapy had a beneficial effect on cognition [48]. The data that has accumulated over time, although it has generated some controversy, suggest that the older, barbiturate-related compounds, such as phenobarbital and phenytoin were more implicated in this process than drugs such as carbamazepine and valproic acid [26]. Thus, one important strategy for enhancing and maximising cognitive abilities in patients with epilepsy is to avoid polytherapy as far as possible, and to attempt monotherapy regimes with non-barbiturate compounds.

Unfortunately, although the era for enthusiastic monotherapy treatment lasted some 20 years, since around 1990 some ten new antiepileptic drugs have become marketed for the treatment of epilepsy, and polytherapy has once again become the name of the game, especially for those with difficult to control seizures, who are likely to come from a population of patients already intellectually compromised on account of the neurological processes underlying their seizures. Interestingly, these newer compounds seem to have less impact on cognitive function than some of the standard treatments, although they have been subject to fewer and less rigorous investigations. Within the spectrum of new antiepileptics, the exception may be topiramate, which, in several studies, has been shown to be associated with cognitive impairments [31,47]. It is of further interest that topiramate seems to be associated with a rather specific localised disruption of cerebral function, namely the induction of an aphasia-like syndrome referred to often as word finding difficulties [32]. These effects seem reversible on stopping the drug.

## 3. Pharmacotherapy of cognitive deficits in epilepsy

People with refractory epilepsy frequently complain of cognitive impairment, and within the broad subject of the neuropsychology of epilepsy, memory deficits represent the dominant complaint in clinical practice, and have been the subject of most neuropsychological investigations [42,46]. Interestingly, several studies have mentioned discrepancies between subjective memory complaints and the objective results of neuropsychological testing [20]. For example, Thompson [46] following a review of the topic, concluded that many patients who have memory complaints often perform within normal limits on standardized memory tests. Therefore, spontaneous complaints of memory difficulties may overestimate the incidence of actual impairment from a neurobiological perspective but, on the other hand, formal neuropsychological studies may underestimate the problem, being non-ecological in their design, usually not measuring performance in everyday situations and thus the real impact on the everyday life of the patients.

Although the immediate and prolonged effects of seizures [10] have a serious impact on cognitive functions, memory impairment is rarely seen as a direct consequence of these factors. However, the role of localized dysfunction, related to on-going epileptic focal activity in specific areas of the brain, is probably one of the key factors for memory impairment [28]. For example, it has now been well demonstrated that even focal interictal spikes as revealed on the EEG can be associated with specific cognitive impairments, including interference with memory [5]. More recent studies suggest an association between the length of seizure history in patients with temporal lobe epilepsy and cognitive decline, although different domains of cognition have not been well studied [23]. Nonetheless, these data emphasize the importance of attempting to get good control of seizures for the benefit of adequate cognition, and changes in drug treatment or improvements of seizure control may have some limited influence on memory impairment. The importance of controlling interictal spikes, especially in younger patients with cognitive and behaviour problems remains a matter of controversy.

It is clear, however, that we need methods to treat memory impairments in epilepsy in clinical practice. Potentially two methods are available: behavioral methods (i.e. training memory and the use of memory aids) [45] and a pharmacological approach. We now review the pharmacological possibilities of treatment. Most of this work relates to memory, but we also refer to some work on attention.

The neuropharmacological approach to memory deficits in epilepsy relates to the axiom that drug treatment is not targeted on structural damage but may be of value in functional impairment, perhaps hastening neuronal recovery from the seizure, improving function of surviving neuronal structures and preventing further

Table 1
Classification of psychostimulants and cognitive enhancers discussed in the text

| |
|---|
| **Non-specific CNS stimulators** |
| Dexamphetamine |
| Methylphenidate |
| Modafinil |
| **Replacement of depleted neurotransmitters** |
| Choline |
| Cholinesterase inhibitors |
| Physostigmine |
| Rivastigmine |
| Donepezil |
| Galantamine |
| **Putative memory modulators** |
| Vasopressin |
| ACTH |
| **Cerebral blood flow enhancers and nootropics** |
| Piracetam |
| Oxiracetam |
| Sabeluzole |

neuronal damage [3]. Theoretically, four main groups of compounds are present in the pharmacological armamentarium of the epileptologist to ameliorate memory functions: non-specific CNS stimulators, substitutes for depleted neurotransmitters, memory modulators and drugs that enhance the cerebral blood flow (Table 1).

## 4. Non-specific CNS stimulators

Non-specific central CNS stimulators, such as amphetamines or caffeine, are found to have a positive effect on cognitive functions, particularly in the presence of attentional dysfunction, slowed cognition and sedation [38,40]. Some CNS stimulants, such as methylphenidate, are considered potentially epileptogenic. However, several studies have demonstrated that generally stimulants can be safely used in patients with brain injury and epilepsy [58] or in epilepsy with co-morbid Attention-Deficit Hyperactivity Disorder (ADHD) [13]. Most positive results have been found for mental speed, arousal activation and associated short term effects, mostly using d-amphetamines [3]. However there are few systematised studies devoted especially to epilepsy. An open-label, non-randomised, 3-month study showed that methylphenidate was safe and effective for patients with epilepsy who suffered sedation, leading to clinically significant improvements in attention, concentration and memory [30].

Modafinil is a newly introduced stimulant that may work by altering the hypothalamic hypocretin-orexin system, and which has been approved for the treatment of excessive daytime sleepiness in narcolepsy. Several studies reported favourable results on attention and memory in patients with brain injury, multiple sclerosis and ADHD [12,43,44,55], but to date we know of no studies in epilepsy. In view of its use in these other CNS disorders, further studies would be useful to investigate the use of modafinil for treating cognitive dysfunction in patients with epilepsy.

## 5. Replacement of depleted neurotransmitters

Replacement of depleted neurotransmitters may have some therapeutic potential in epilepsy in the future but, so far, this approach has not been well explored, as it has in other specific syndromes, such as Alzheimer's disease, with for example the reinforcement of the cholinergic system [3]. The rationale for this approach is analogous to dopamine supplementation in Parkinson's disease, and cholinergic replacement for Alzheimer's disease is based upon underlying neuropathological findings and represents, at the present time, the main therapy. However, the theoretical basis of memory disorders in epilepsy is less understood and the potential neurochemical mechanisms are unknown.

In animal models, seizure-induced memory impairment is reduced by choline supplementation before or after status epilepticus [22]. Holmes et al. [22] investigated the effects of pre- and post-natal choline supplementation in rats with memory deficits associated with status epilepticus induced with kainic acid. Two weeks following the kainic acid-induced status, rats underwent testing of visual-spatial memory using the Morris water maze test. Rats receiving choline supplementation performed better in the water maze than the deficient and control groups. The authors suggested that choline supplementation can protect rats from memory deficits induced by status epilepticus.

Clinical studies have reported a useful effect of the cholinesterase inhibitor donepezil in treating memory dysfunction in patients with brain injury [53,54], and one open label study of donepezil in patients with focal epilepsy suggested improvement in certain aspects of memory [14]. In this 3-month, open label study of 5–10 mg donepezil in 18 patients, the authors observed on a battery of neurocognitive tasks, no change in attention, visual sequencing, mental flexibility or psychomotor speed, but did report a significantly greater number of words recalled across learning trials. Seizure frequency worsened in 2 of the 18 patients but these changes in seizure frequency were not statistically significant. Although seizures, as a potential side effect of cholinesterase inhibitors, might be of special concern in patients with epilepsy (and in the patient information sheet, convulsions are mentioned in overdose), seizures were never considered a significant adverse event in pre-marketing trials of donepezil [38]. To our knowledge none of the other cholinergic agonists have been examined in patients with epilepsy.

## 6. Putative memory modulators

The administration of putative memory modulators for neurological disorders has been proposed using several types of neuropeptides, such as vasopressin, and of the 20 or more potentially active CNS peptides ACTH, vasopressin and opioid peptides have been studied the most. Although the results of studies with experimental models were encouraging, clinical trials showed evidence of some efficacy on memory, but only in patients with diabetes insipidus [24] and the benefits of neuropeptides as memory enhancers has been seriously questioned [8]. To our knowledge there are no studies using these molecules in epilepsy.

## 7. Cerebral blood flow enhancers and nootropics

Several types of drugs are available that claim to increase cerebral blood flow or to enhance CNS cellular metabolism. This may be a promising approach for the treatment of memory disorders in epilepsy, because some trials with these drugs have shown protective properties against amnesia in animal models, such as the electroshock model or that of induced cerebral hypoxia, that may have some relevance for human epilepsy [25,36]. Lagergren and Levander [25] induced artificial hypoxia through a controlled reduction of heart rate in rats. Piracetam, the first drug to be developed in this class, had little effect on performance at normal heart rate, but significantly decreased the cognitive deterioration caused by the hypoxia when the heart beat was reduced. The same mechanism may be assumed for causes other than hypoxia since piracetam and related nootropics (such as oxiracetam) also have been shown to protect against other animal models of amnesia [1].

In epilepsy, this class of drugs may be used as add-on treatment against the sedative side effects of the antiepileptic drugs, one of the important causes of cognitive blunting which may be clinically relevant, especially if changes in a patient's antiepileptic drug regimen are not possible. Some positive results with oxiracetam have been reported in healthy volunteers normalizing diazepam-induced memory changes [15] and with other piractam related molecules in recovery from the amnestic effects of drug intoxication [3].

A subclass of drugs claimed to enhance cerebral blood flow are referred to as the nootropic drugs, which not only act as vigilance-enhancing psychostimulants, but may also influence specific cognitive targets [16, 17,36]. The mechanism of action of this class of drugs is assumed to have a selective effect on brain integrative functions, with no specific or identified alteration in neuronal excitability or neurotransmitter activity [16,17]. Several specific cognitive targets for these drugs have been proposed. Some studies suggest that cognitive improvement with the nootropic drugs may be the results of a general psychostimulant effect on arousal and vigilance [57]. Their quantitative-EEG profile is very similar to that of general CNS stimulating drugs such as amphetamine, revealing an alpha rhythm-enhancing effect, an increase in the average EEG frequency and a decrease in delta activity [15,52, 57].

Interestingly, a specific effect on verbal processing has been suggested. Piracetam, for example, has been studied in children with dyslexia. This approach was based on the observation in early studies, that substantial improvement of verbal sequential processing occurred in children with learning disorders after administration of piracetam [9,39]. In some cases, improvement of reading disability was found but only in limited areas and most effects were weak [7,56]. More pronounced changes were shown in enhanced short term memory, especially with tests of verbal information, as indicated by word recall. Therefore, short-term memory deficits may be a more promising target for this class of drug treatment. This is in line with other clinical studies on piracetam, such as the studies of Helfgott et al. [18,19] and Levi and Sechi [27] or studies of related drugs such as etiracetam [37] or aniracetam [35], all of which imply some effect of this class of drug on cognition in those with varying degrees of handicap.

Some authors have evaluated the effect, in patients with epilepsy, of the nootropic drugs on memory functions and severe memory impairment. An early trial involved oxiracetam, a pyrrolidinone derivative, structurally related to a cyclic form of gamma-amino-beta-hydroxybutyric acid [51]. For oxiracetam, the same mode of action is proposed as for piracetam, only with more distinctive effects on memory [4], especially in elderly people with organic brain syndromes [21], in patients with multi-infarct and primary degenerative dementia [11], and in a heterogeneous group of dementia patients [29]. A randomized double-blind placebo-controlled 12 week study of oxiracetam (800 mg three times daily) vs placebo in people with epilepsy showed significant improvements in the neuropsychological profile of the patients, suggesting enhancement of processing speed as well as of language comprehension [1]. The authors observed again the greatest improvement for language. No specific improvements in memory were found. These findings are in keeping with reports about piracetam-induced effects on verbal processing [7,56] and with studies that found primary effects on alertness and vigilance [3,57]. However, these results should be cautiously interpreted because in most of the tests, the noted improvements were quite weak.

A related compound is levetiracetam, which has now been successfully introduced for the management of epilepsy. It mechanism of action as an antiseizure agent is unknown, and there are few studies on its effects on cognition. In a single case report, this compound was shown to improve stuttering [34]. Aldenkamp and his group have studied the effects on cognition of levetiracetam in patients with epilepsy, but their single-blind study of the drug as add on therapy after one week showed no discernable effect [33].

Another trial involved sabeluzole, a benzothiazol derivative that also has been shown to be an effective protector against several types of hypoxia in animal models. The results in normal volunteers showed that sabeluzole ameliorated both learning and memory recall, in conditions of age-related mild hypofunction [6]. Consolidation of information appeared to be more efficient and recall of verbal information from long-term memory was improved even one week after withdrawal of the drug [6]. Further experiments consistently have shown most of the positive effects in subjects with initially relatively poor performance, such as in a subgroup of patients with the lowest scores on baseline memory tests [49]. However, the evidence, taken together, is not clear as to whether sabeluzole improves memory function per se or reactivates or restores less specific impaired cognitive function. Although the mechanism of action of sabeluzole is not completely clear, its role in improving fast axonal transport and the effect on voltage-dependent outward K+ currents may play a role in its cognitive effects [2].

A randomized, double-blind placebo-controlled parallel-group study of 38 patients with epilepsy, assigned to a 12 week treatment with either sabeluzole or placebo showed a number of responders (patients with an improvement that was more than 1 SD on at least three of the memory tests) in the sabeluzole group (64%), which was nearly twice the number of responders in the placebo group (36%) [2]. This suggested a potentially clinically relevant effect of sabeluzole in the memory problems associated with epilepsy, and its effect on long-term verbal memory tests may represent a specific drug effect.

## 8. Conclusions

It is now acknowledged that disorders of cognitive function, especially memory complaints, are frequent in patients with epilepsy. Although memory problems are interlinked with the known underlying neurobiology of epilepsy in many cases, namely hippocampal sclerosis, and are likely influenced by ictal and interictal neurophysiological disturbances of neuronal circuitry related to memory and attention, it is clear that antiepileptic drugs can further interfere with cognition, and some are more potent in this regard than others. However, in spite of this being such an important clinical area, little research has been done on the neuropharmacology of cognitive rehabilitation in epilepsy, an area in need of urgent study.

Although the evidence is weak, the role of some drugs in cognitive rehabilitation of patients with epilepsy seems to be promising. We note in particular the work with piracetam and its derivatives, and with sabeluzole, which may provide clues to the trials of other related chemicals. Two related areas, the molecular biology of synaptogenesis and the role of apoptosis represent prominent targets for research in the field of neuroprotection, and it is known that a number of psychotropic agents enhance both synaptogenesis and neurogenesis [50]. Such studies have not so far yielded comparable data for antiepileptic drugs, but the role of neuroprotection in preventing long-term deterioration of function and disability in chronic disorders, such as epilepsy, is an important area of future research. Further, a better understanding of the pathophysiology of cognitive deficits in epilepsy is the basis for the future development of specific drug therapies.
